# Editorial

**Published:** 1841-01-16

**Authors:** 


THE MEDICAL EXAMINER.

PHILADELPHIA, JANUARY 16, 1841.

### INCREASE OF MEDICAL JOURNALS.

The multiplication of medical journals in the United States has been a natural concomitant of the general development of the resources of the country. This increase in the number of journals is a gratifying index of an increasing demand for information, which, in its turn, it cannot fail to foster and promote. We have always hailed with pleasure the appearance of new medical periodicals, with prospects of sustaining themselves—believing that wherever they are scattered, they must exercise a fertilizing influence, as well generally as locally. So far, too, from interfering with established interests, we are of opinion, that every additional well-conducted journal *creates* readers not only for itself, but for those already in the field. With our immense continent, of varied climates and diseases, scarce a hamlet of which is unprovided with a well-educated physician, is it not too much to assume the solitary journal which a few years occupied the ground, or even the half dozen others which have since sprung up, to be satisfactory expositors of the accumulation of facts and opinions which such resources ought to furnish, or sufficient stimulus to the due development of these resources. To multiply the channels of information, is to promote the advancement of knowledge,—the attempt to restrict them, is as futile as it is illiberal.

Most of our readers will feel surprise at learning that something of this narrow spirit lurks in a quarter where more enlarged views might have been looked for. Our respected cotemporary, the American Journal of the Medical Sciences, betrays evident uneasiness at the growth of journals around it; and while itself offering the best practical commentary upon the good effects of competition, is weak enough to evince symptoms of a spirit of monopoly. The January number of the Journal, the first of a fresh series, appears in a new and showy dress, and is heralded by promises of "such improvements in the plan, as the enlarged experience of the editor and his able colleagues may suggest." These efforts at improvement are the palpable and wholesome results of corresponding efforts elsewhere—in fact of the very multiplication of journals, which the parties interested in the American Journal seem to be directly and indirectly attempting to interfere with. We are informed by the Western Journal of Medicine and Surgery, that—

"In the course of last summer, a distinguished Philadelphia physician, warm in his friendship toward the American Journal, and interested in its standing, passed a few days of sojourn in the city of Louisville. And while here, he made to some of the physicians of the place, a direct proposal to the following effect. He expressed a wish that no medical journal might be henceforward published west of the mountains; but that western physicians should write only for the "American Journal," in order that the concentrated result of the observation, reflection, experience, and every other form of research and information, of the whole American faculty of medicine,—a faculty whose resources are furnished by a region more extensive than Europe, and embrace all that concerns the health of sixteen or eighteen millions of people,—in order, we say, that this mighty mass of professional intelligence might be proclaimed to the world by a single oracle—and that oracle the American Journal!"

On such a proposal, in the words of the Western Journal, comment would be lost. Of a piece with it, however, is the tone of the circular prefixed to the "new series." In the self-puffing style of some of the penny papers of the day, it is asserted that " the American Journal is regarded by the great mass of the American medical profession as their representative, and as such is received and quoted abroad!" We are not disposed to disturb our cotemporary in his self-complacency, but we must be excused for reminding him that even of his able band of collaborators many are writers for other journals. Certainly, among them, Chapman, Coates, Gibson, Harris, Jackson, Horner, Pennock,—nomina haud ignota,—have contributed "more elaborate and comprehensive articles" for the Examiner than for the American Journal, during the period of the existence of the former. We cheerfully, however, bear testimony to the usefulness and merit of our respectable cotemporary, and shall rejoice in his prosperity and prospects of continued success. We wish him only better taste and temper, and suggest that the next time he puffs his quarterly, he leave to others to point out "the great advantages it possesses over a weekly journal of small size."

